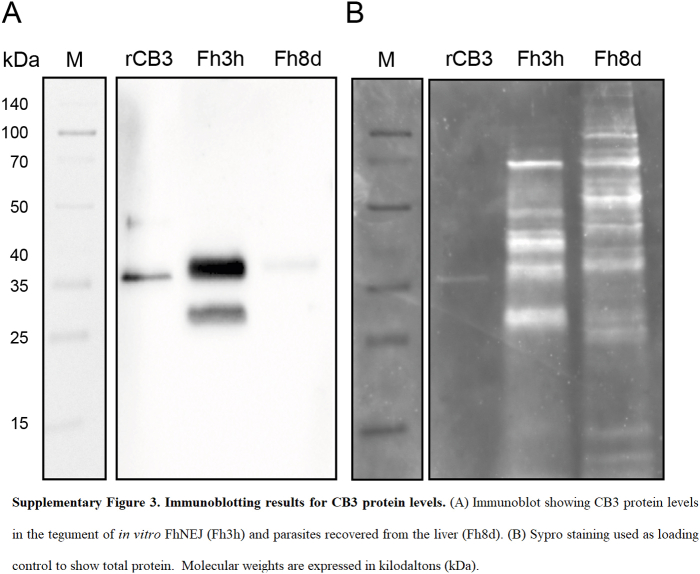# Deciphering the Proteomic Profile of the Parasite *Fasciola hepatica* After Initial Vertebrate Host Infection Reveals New Insights Into Its Early Stages

**DOI:** 10.1016/j.mcpro.2025.101005

**Published:** 2025-05-31

**Authors:** Marta López-García, Krystyna Cwiklinski, David Becerro-Recio, María Teresa Ruiz-Campillo, Verónica Molina-Hernández, José Pérez-Arévalo, Álvaro Martínez-Moreno, Javier González-Miguel, Mar Siles-Lucas

**Affiliations:** 1 Laboratory of Helminth Parasites of Zoonotic Importance (ATENEA), Institute of Natural Resources and Agrobiology of Salamanca (IRNASA-CSIC), Salamanca, Spain; 2 Institute of Infection, Veterinary and Ecological Sciences, Faculty of Health and Life Sciences, University of Liverpool, Liverpool, United Kingdom; 3 Departamento de Anatomía y Anatomía Patológica Comparadas y Toxicología, UIC Zoonosis y Enfermedades Emergentes (ENZOEM), Facultad de Veterinaria, Universidad de Córdoba, Córdoba, Spain; 4 Departamento de Sanidad Animal (Área de Parasitología), UIC Zoonosis y Enfermedades Emergentes EN-ZOEM, Facultad de Veterinaria, Universidad de Córdoba, Córdoba, Spain

**Keywords:** comparative proteomics, early stages, *Fasciola hepatica*, mouse infection

## Abstract

The migration of the trematode parasite *Fasciola hepatica* within its vertebrate host following infection by ingestion of the metacercariae represents a critical event in its establishment and survival. The early stages of infection, during which *F. hepatica* crosses the intestinal barrier and advances to the liver through the peritoneum, initiate changes in the parasite that drive its development from a free-living state on pasture to an obligate blood-feeding parasite. Using an *in vivo* mouse model, this study explores the proteomic changes in the parasite as it crosses the intestinal barrier and migrates to the peritoneal cavity (24 h post infection (p.i.)) and liver parenchyma (8 days p.i.). This model was coupled with SWATH-MS, enabling a comparative evaluation of parasite protein abundance during the early stages of infection. We identified a total of 1180 *F. hepatica* proteins from three developmental time points: newly excysted juveniles (FhNEJ) at 3 h post excystment *in vitro*, and parasites collected *in vivo* at 24 h and 8 days p.i., separated into two different parasite compartments (somatic and tegumental). These extracts exhibited differentially expressed proteins (DEPs) across the analyzed time points, with 274 and 463 differentially expressed proteins identified in parasites obtained at 24 h and 8 days p.i., respectively. Our findings further highlight the adaptations *F. hepatica* undergoes within the first week of infection, including a shift toward anaerobic metabolic pathways, induction of signal transduction pathways involved in growth, and enrichment of crucial cysteine peptidases associated with feeding and immunomodulation. This study represents the first in-depth proteome analysis of parasites recovered 8 days into infection, adding to the wealth of molecular data available for *Fasciola* spp. to enhance our understanding of early host–parasite interactions. These data are crucial for the development of future *in vitro* models of fasciolosis and for identifying vaccine candidates targeting the early parasite stages.

## Highlights

• This is the first study to reveal early proteomics of *F. hepatica* in a mouse model.
• SWATH-MS quantified 1644 proteins; 71.78% were stage-specific and parasite-derived.
• Proteomic changes showed 274 and 463 proteins altered at 24 h and 8 days p.i.
• Key protease profiles were defined exclusively in early infection by *F. hepatica*.
• Findings show metabolic shifts and evasion tactics of *F. hepatica* during migration.

## Graphical Abstract

**Figure:**
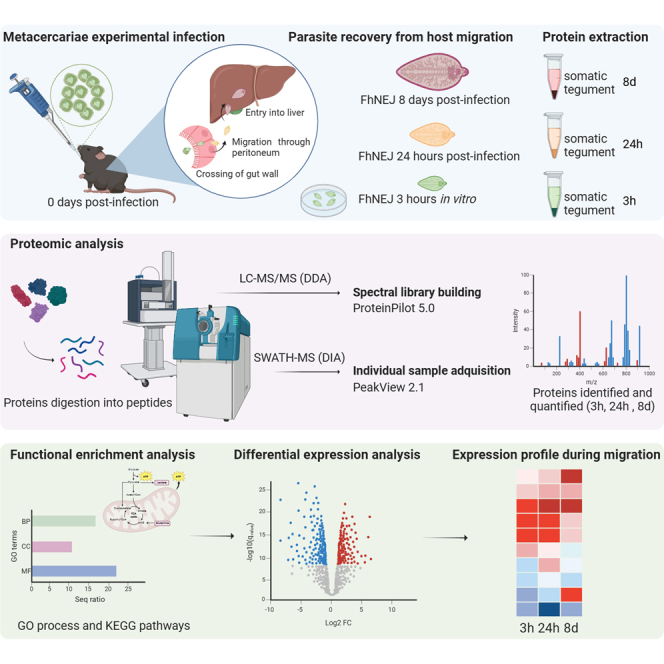

## In Brief

This study analyzes the proteomic changes in *Fasciola hepatica* during the early stages of infection in a mouse model. Using SWATH-MS, 1180 parasite proteins were identified at different stages, highlighting differentially expressed proteins at 24 h and 8 days post infection. The results reveal key adaptations in anaerobic metabolism, growth signaling, and enrichment of cysteine peptidases, providing crucial insights for developing models and vaccines targeting early stages of fasciolosis.

The trematode *Fasciola hepatica* is the primary causative agent of fasciolosis, a foodborne parasitic disease with a worldwide distribution (1). This parasite has a remarkable capacity to thrive within different definitive hosts, mainly large-size herbivorous species (2). Consequently, the livestock industry is confronted with a significant challenge, resulting in annual economic losses estimated at $3 billion globally (3). In addition, the zoonotic potential of *F. hepatica* has a considerable public health impact as a neglected tropical disease (4), affecting up to 17 million people across more than 80 countries (5, 6).

The challenge in managing fasciolosis stems from the complexity of the *F. hepatica* biological cycle, which, in the vertebrate host, begins with the ingestion of aquatic vegetation contaminated with the infective stage of the parasite, the metacercariae. Upon reaching the small intestine, the metacercariae undergo excystment, releasing the newly excysted juveniles (FhNEJ), which penetrate the duodenal wall within 3 h after infection (7). The juvenile parasites then embark on a migratory journey through the peritoneal cavity toward the liver, where they arrive typically between 4- and 10-days post infection (p.i.), persistently traversing through the liver parenchyma for several weeks until they reach the biliary ducts and reach maturity (8). These early migration events have been regarded as the “point of no return” in fasciolosis (9), as controlling the infection at this stage could prevent the parasite from reaching its definitive location in the biliary ducts. As *F. hepatica* migrates, it undergoes metabolic adaptations to the changing environments of host tissues. During the early stages of infection within the definitive host, the parasite primarily relies on aerobic metabolism, generating ATP through glycolysis and oxidative phosphorylation. However, as it progresses through the liver parenchyma, a metabolic shift occurs, gradually favoring acetate production while still requiring oxygen for NADH reoxidation. Upon reaching the bile ducts, *F. hepatica* fully transitions to anaerobic metabolism, relying on fermentation to sustain ATP production. This progressive shift from aerobic to anaerobic energy metabolism highlights the parasite’s metabolic plasticity, which is essential for its survival within the host (10). In addition, the parasite’s development is also supported by a complex proteolytic system composed of cysteine proteinases from the cathepsin and legumain families. *F. hepatica* expresses specific types of proteases depending on the host tissue it locates, along with the necessary inhibitors to maintain proteolytic balance (11). This system plays a crucial role in tissue invasion, migration, nutrient acquisition, and immune evasion (12). These adaptations are reflected in fluctuating protein expression throughout the parasite’s life cycle, ultimately complicating control strategies (13). Understanding the molecular events that the parasite undergoes while advancing through the tissues of the vertebrate host is therefore critical for the development of future control strategies.

Although advances have been made in the development of *in vitro* (14) and *ex vivo* models of fasciolosis (7), *in vivo* animal models remain essential for the comprehensive study of parasite migration and development, as they provide *in situ* host environmental cues (13). The combination of these models with the current state-of-the-art technologies have yielded comprehensive molecular insights, taking advantage of the increasingly established omics approaches in the field, providing new knowledge on the virulence, growth, and development of the parasite in the mammalian host (15). Extensive omics datasets are currently available, including a range of proteomic studies of the excretory-secretory (ES) proteins, and somatic and tegumental proteomes (16, 17, 18), facilitating the investigation of *F. hepatica* infection and invasion dynamics. However, the studies of early stage infection have mainly focused on *in vitro* FhNEJ parasites recovered post excystment and *in vivo* parasites recovered 21 days p.i. As a result, a significant knowledge gap remains regarding the *in vivo* mechanisms that occur after the parasite traverses the gut wall and establishes itself in the liver within the first week of infection.

The objective of this study was to characterize the proteomic expression profile of *F. hepatica* parasites as they migrate from the small intestine to the liver through the peritoneum in the vertebrate host. We used Sequential Window Acquisition of All Theoretical Mass Spectra (SWATH-MS), which facilitated the simultaneous identification and quantification of thousands of parasite proteins, enabling the assembly of an in-depth and precise molecular profile of the early stages of infection within the host. This analysis elucidated the proteins involved in the metabolism, growth and evasion immune mechanisms that facilitate the *F. hepatica* migratory route *in vivo* within the mouse host.

## Experimental Procedures

### Ethics statement

Experimental animals were used in compliance with the current European regulations outlined in Directive 2010/63/EU, ensuring their protection. Ethical guidelines for animal housing, sacrifice, and experimentation were strictly followed. Experimental procedures were approved by the Ethical Animal Experimentation Committee of the University of Córdoba (project no. 2021PI/22) and by the Junta de Andalucía (Spain) (project no. 10/11/2021/175).

### Experimental Design and Statistical Rationale

Proteins from FhNEJ after 3 h of *in vitro* culture from a previous study (7) were compared to proteins recovered from parasites obtained *in vivo*, specifically from the peritoneal cavity of mice at 24 h p.i. and from the liver parenchyma at 8 days p.i. The protein extraction process was carried out separately for the somatic and tegumental fractions, both of which were analyzed using SWATH-MS proteomics. For the proteomic analysis, well-established and validated analysis pipelines were employed to ensure accurate and reliable results. The choice of cutoff values for statistical significance was determined based on the nature and characteristics of the respective data sets. Detailed descriptions of the specific analytical and statistical methodologies, including data processing, normalization, and statistical testing, are outlined in detail in the respective method sections below.

### Set-Up of an *In Vivo* Mouse Infection Model for the Collection of Early Stages of *F. hepatica*

C57BL/6 mice (Charles River Laboratories) were housed at 22 ± 3 °C, 50 to 60% of relative humidity and 12 h light/dark cycle in facilities of the Experimental Animal Service of the University of Córdoba (Spain). After 1 week of acclimatation period, mice were orally infected with 200 *F. hepatica* metacercariae of Italian strain (Ridgeway Research Ltd) per animal. The infection was carried out with resuspended metacercariae in 20 μl of water, administered using a pipette with a yellow tip previously washed in sterile PBS + 0.01% Triton X-100 to prevent the metacercariae from sticking to the plastic tip and trimmed 0.5 cm to ensure their direct delivery into the posterior part of the mouse's mouth. After infection period, sacrifice was done by euthanizing mice by cervical dislocation after the loss of consciousness using a CO_2_ chamber for 2 to 3 min.

For the recovery of juvenile worms from the peritoneum, an incision was made to access the cavity, and a pipette tip was inserted to collect the peritoneal fluid containing the parasites. In addition, three washes of the peritoneal cavity were performed with 1 ml of PBS to collect the remaining parasites. The viability of the parasites was determined by assessing their motility on a plate containing PBS under a stereomicroscope. To obtain parasites migrating through the liver parenchyma, complete livers were dissected with tweezers under a stereomicroscope to collect the juvenile worms. In both cases, viable *F. hepatica* juvenile worms were counted, measured, and finally collected in Eppendorf tubes with PBS. FhNEJ cultured *in vitro* for 3 h and fixed in 4% paraformaldehyde, obtained from a previous study (7), were used as a comparative group for measurements.

To determine the optimal time points for parasite recovering from the peritoneal cavity and liver parenchyma, five time points were examined: 6 h, 24 h, 48 h, 6 days, and 8 days p.i. (19). For the proteomic analysis, parasites recovered from the peritoneum at 24 h p.i. and the liver at 8 days p.i. on three respective independent days were considered biological replicates.

### Parasite Protein Extraction

Enriched protein fractions of the *F. hepatica* somatic and tegumental proteins were extracted from parasite samples using the methodology described by Becerro-Recio *et al*. (2022) (7). Briefly, parasites were centrifuged at 300*g* for 5 min and washed twice with sterile PBS. The samples were incubated in PBS containing 1% Nonidet P-40 (Sigma-Aldrich) and a cocktail of serine, cysteine, aspartic, thermolysin-like protease, and aminopeptidase inhibitors (Sigma-Aldrich) (1x), at a ratio of one parasite per microliter, to extract the tegumental fraction at room temperature (RT) with gentle stirring for 30 min. After incubation, the samples were centrifuged at 300*g* for 5 min, and the resulting supernatant, containing the enriched tegumental protein fraction, was stored at −80 °C. The pellet was resuspended in 1:1 radioimmunoprecipitation assay buffer (Sigma-Aldrich) and incubated with protein inhibitors (Sigma-Aldrich) for 15 min on ice. Subsequently, the protein samples underwent ultrasound sonication (4 cycles of 30 s each). The samples were then centrifuged at 1300*g* for 10 min, and the resulting supernatant, containing the somatic fraction, was stored at −80 °C.

### Mass Spectrometry Analysis of Parasite Proteome

Protein samples were quantified using a detergent-compatible kit (Protein Quantification Assay) (Macherey-Nagel) according to the manufacturer's instructions. Sample preparation involved concentrating the samples on a gel to obtain approximately 10 μg of total protein for the creation of spectral libraries, which also removed the Nonidet P-40 from the tegumental samples to make them compatible with subsequent trypsin digestion. Gel electrophoresis was carried out using an AnyKD precast gel (Bio-Rad) at 200V for 5-20 min, after which the gel was fixed in a solution of 40% ethanol and 10% acetic acid for 1 h, followed by staining with colloidal Coomassie (Bio-Rad) for an additional hour. Digital imaging of the gel was conducted using an Image Scanner (GE Healthcare). The samples were digested using sequencing grade trypsin (Promega), following the previously described protocol by Shevchenko *et al*. (1996) (20) with the modifications outlined below. The digestion process was stopped by adding 1% trifluoroacetic acid (TFA), after which a dual extraction was performed using acetonitrile (ACN). The resulting peptide solutions were dried using a rotary evaporator and reconstituted in a solution of 2% ACN and 0.1% TFA, resulting in a final protein concentration of 0.3 μg/μl.

Liquid chromatography-tandem mass spectrometry analysis was carried out using an Ekspert nanoLC 425 (Eksigent) with a nanoESI qQTOF mass spectrometer (6600plus TripleTOF, ABSCIEX). A 5 μl peptide mixture was desalted on a trap column (3μ C18-CL 120 Å, 350 μm × 0.5 mm; Eksigent) using 0.1% TFA at 5 μl/min for 3 min. The peptides were then transferred to an analytical column (3μ C18-CL 120 Å, 0.075 μm × 150 mm) (Eksigent) prepped with 5% ACN and 0.1% formic acid (FA). Elution was carried out over 60 min with a linear gradient from 7% to 40% B in A (A: 0.1% FA and B: ACN, 0.1% FA) at a flow rate of 300 nl/min. All samples were ionized at 2.8 kV, utilizing a data-dependent mode. MS1 scans covered a range of 350 to 1250 m/z for 250 ms, while MS2 scans covered a range of 100 to 1500 m/z for 150 ms in high sensitivity mode.

SWATH liquid chromatography-tandem mass spectrometry analysis was carried out on 5 μl of each sample desalted on a NanoLC column (3μ C18-CL, 75 μmx15 cm) (Eksigent) at 0.1% TFA and a flow rate of 2 μl/min for 10 min. The peptides were loaded onto an analytical column (3μ C18-CL, 75 μm x 12 cm) (Nikkyo) that was equilibrated with 5% ACN and 0.1% FA. Elution was performed using a 90-min linear gradient from 5% to 40% B (A: 0.1% FA and B: ACN, 0.1% FA) at a flow rate of 300 nl/min. Samples were analyzed using a nanoESI qQTOF mass spectrometer (6600 plus TripleTOF, ABSCIEX) in SWATH mode (data-independent acquisition). A 0.050-s TOF MS scan covering 350 to 1250 m/z was conducted, followed by 0.080-s product ion scans across 37 predefined windows (3.05 s/cycle), employing 15 Da window widths from 450 to 1000 Da. Analyses were done at the Proteomic Service from the University of Valencia.

### Data Search

ProteinPilot version 5.0 software (SCIEX) was used to generate peak lists from 6600 TripleTof.wiff files for library construction, including the.wiff files of the FhNEJ obtained *in vitro*, available from the study by Becerro-Recio *et al*. (2022) (7) in the PRIDE repository (accession number PXD033952) (21). These samples corresponded to somatic and tegumental extracts from FhNEJ obtained after 3 h of *in vitro* incubation (7), whose mass spectra (.wiff files) were incorporated into the proteomic analysis as a comparative group.

Searches were conducted against the predicted proteome of *F. hepatica* (PRJEB25283, https://parasite.wormbase.org/Fasciola_hepatica_prjeb25283/Info/Index), and proteome of *Mus musculus* (https://www.uniprot.org/proteomes/UP000000589), appended to the cRAP contaminant database (https://www.thegpm.org/crap/), which included 65,189 proteins and 130,378 proteins including decoys for false discovery rate (FDR) calculation, using the Paragon algorithm with trypsin specificity, iodoacetamide cys-alkylation, and unrestricted taxonomy settings. FDR calculations followed the methodology of Shilov *et al*. (2007) (22). The Pro Group algorithm grouped proteins based on observed peptides sharing physical evidence, excluding unobserved sequence regions and selected proteins that had at least two identified peptides and <1% FDR for subsequent analysis. SWATH.wiff files were processed using PeakView 2.1 (SCIEX). Retention times were aligned across various samples by utilizing seven peptides from abundant proteins across all samples, ensuring they covered the entire retention time range of the chromatography analysis. Normalization occurred by dividing each protein's area by the total sum of areas from all quantified proteins.

### Bioinformatics Analysis

Principal component analysis (PCA) was performed using the *FactoMineR* (23) and *factoextra* (24) packages in R software (version no. 4.1.0). The analysis included data loading, PCA implementation and subsequent analysis extension to fully visualize and understand the data structure. OmicsBox 3.1.2 software was used to annotate *F. hepatica* proteins via NCBI blastp and EMBL-EBI InterPro algorithms against the flatworm database (taxa: 6157, Platyhelminthes) to derive Gene Ontology (GO) terms (25) encompassing biological process, molecular functions, and cellular components categories, and Kyoto Encyclopedia of Genes and Genomes (KEGG) pathways. Manual curation of the KEGG pathways was also carried out using the KEGG Automatic Annotation Server (26). Manual curation of the *F. hepatica* cysteine peptidases (cathepsin and legumain proteins) and protease inhibitors was carried out based on available *F. hepatica* genome data (27, 28) and previous characterization of these gene families (11, 15).

Clustering analysis of the mean proteomic abundance values from three replicates was performed by R software on the whole proteome. GO enrichment analysis was conducted using Fisher's Exact Test (filter value of 0.05, *p*-value adjustment and Benjamin–Hochberg FDR method as the multiple *t* test) for each protein cluster (29) in order to extract GO terms that showed significant overrepresentation or underrepresentation compared to all identified proteins, within the somatic or tegument enriched fraction. Protein expression profiles, based on SWATH-MS abundance values were visualized as heatmaps created by the R package pheatmap (30).

Statistical analysis was conducted on expression values by performing quantitative examination after calculating the Log2 of each identified protein using GraphPad Prism 9.5.1 software. Differentially expressed proteins (DEPs) were determined between the *in vitro* FhNEJ group and *in vivo* samples (parasites obtained at 24 h and 8 days p.i.) via Student's *t* test followed by post hoc corrections by the method described in Benjamini *et al*. (2006) (31). Proteins displaying a q-value below 0.01 were deemed to be differentially expressed and were presented as volcano plots using the *ggplot2* package (32) in R software.

### Immunoblotting

Tegumental extracts from FhNEJ cultured *in vitro* for 3 h (obtained in a previous study (7) and stored frozen in our laboratory) and parasites collected from the liver at 8 days p.i. were selected as representative groups to validate the manual annotation carried out for cathepsin peptidases in the bioinformatic analyses. In this way, these extracts were concentrated using filters with a nominal molecular weight limit of 5 kDa (Merck Millipore) by centrifuging at 14,000*g* for 30 min at 4 °C. Protein concentrations were determined by the Pierce BCA Protein Assay kit (Thermo Fisher Scientific). Both samples and a recombinant version of cathepsin B3 from *F. hepatica* (rFhCB3, 0.50 μg/ml) kindly provided by Prof. John Dalton (University of Galway) (33) were added to Laemmli buffer, boiled for 5 min to denature the proteins, resolved in 12% SDS-PAGE gel and electrotransferred onto a nitrocellulose membrane as described in Cañada-García *et al*. (2023) (34). Total protein staining was then performed with SYPRO Ruby (Thermo Fisher Scientific) according to the manufacturer’s instructions. Membranes were subsequently incubated in blocking buffer (2% bovine serum albumin in PBS-0.05% Tween-20 (v/v) (PBST)) for 1 h at RT. The primary antibody, a polyclonal mouse-anti-*F. hepatica* cathepsin B3 obtained against the rFhCB3 (Davids Biotechnologie) was diluted 1:5000 in PBST and added to the blot overnight at 4 °C. After three washes in PBST, the secondary antibody, horseradish peroxidase labeled goat to mouse-anti-IgG (Sigma-Aldrich) (1:2000) was added to the membrane and incubated for 1 h at RT. The blot was then processed using the enhanced chemiluminescence method (Bio-Rad) on a ChemiDoc MP Imaging System (Bio-Rad).

## Results

### Set-Up of an *In Vivo* Mouse Infection Model for the Collection of Early Stages of *F. hepatica*

**Fig. 1:**
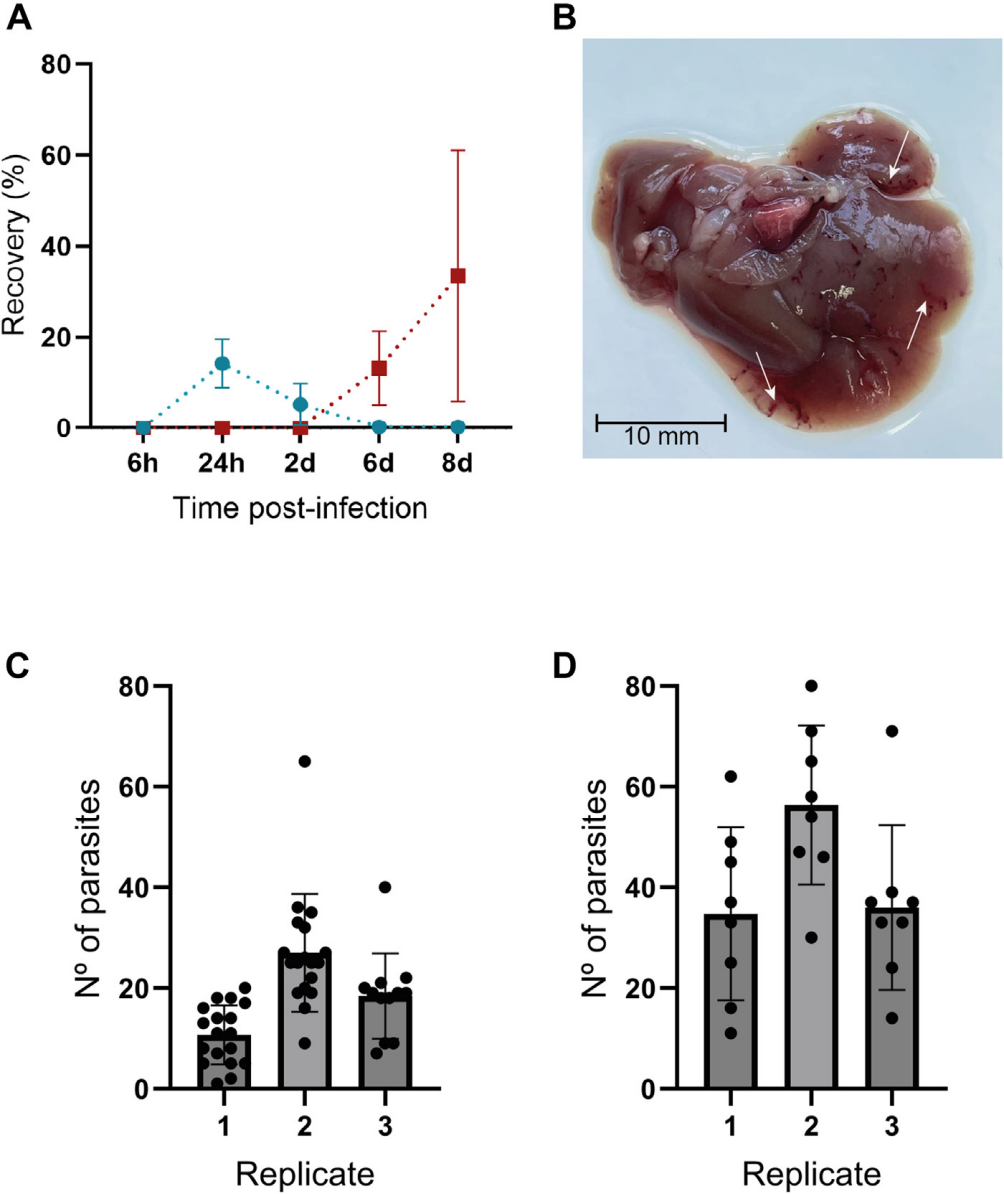
**Set up of an *in vivo* model of *Fasciola hepatica* infection in mice.***A*, *blue circles* represent parasites collected from the peritoneal cavity, while *red squares* represent parasites collected from the liver tissue. Each time point contains data from two mice, with error bars representing the standard deviation (SD). *B*, representative liver image of one of the infected mice after 8 days of infection. Tracks of the migrating parasites are shown (*white arrows*). *C*, bar graph showing the number of parasites recovered from the peritoneal cavity 24 h post infection, with error bars representing the standard deviation (SD). Parasites were collected from 48 mice. *D*, bar graph showing the number of parasites recovered from the liver parenchyma 8 days post infection, with error bars representing SD. Data were collected from 24 mice. “Replicate” refers to bath of parasite recoveries performed on independent days under the same experimental conditions.

We investigated the timing of *F. hepatica* migration through the mouse peritoneum and liver following oral infection using the *in vivo* infection established in this study. After obtaining and counting the number of parasites at each location at different times p.i., the highest percentage of parasites in the peritoneum was found at 24 h p.i., with a mean of 14.25% ± 5.30% for the administered/collected parasite ratio. This was followed by a gradual decrease at subsequent time points, with a mean of 5.25 ± 4.60% at 2 days p.i. Parasites remaining in the peritoneum at 6 and 8 days p.i. exhibited a lack of movement (mean of 0.25% ± 0.35%). Juvenile parasites were found in the liver tissue at 6 days p.i. (13.25% ± 8.13%), reaching their peak at 8 days p.i., with a mean of 33.50% ± 27.58% for the administered/recovered parasite ratio (Fig. 1*A*). Liver examination of mice euthanized at 8 days p.i. revealed clear evidence of parasite migratory tracks within the liver parenchyma (Fig. 1*B*).

**Supplemental Fig. S1:**
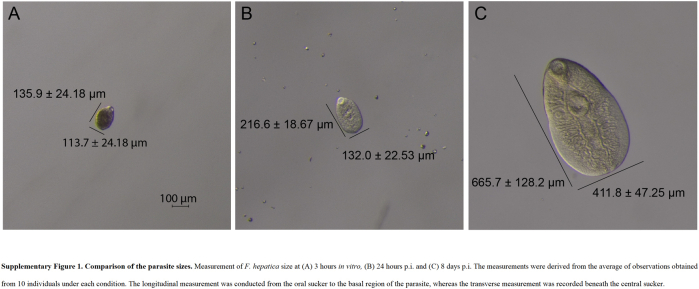


Based on these results, a second experiment was conducted to collect parasites at 24 h and 8 days p.i. *F. hepatica* parasites were recovered from the peritoneal cavity at a rate of 9.38% at 24 h p.i., with a mean number of parasites per mouse of 10.72 ± 5.86, 27.00 ± 11.67, and 18.42 ± 8.51 for each of the three experimental replicates, respectively. Each replicate corresponds to the total number of mice used on each of the three independent experimental days (Fig. 1*C*). In the liver parenchyma, the recovery rate was 21.19% at 8 days p.i., with mean parasite counts of 34.75 ± 17.19, 56.38 ± 15.79, and 36.00 ± 16.40 in each replicate (Fig. 1*D*). The viability of the collected parasites was assessed by observing their motility, as shown in Supplemental Videos S1 and S2. Parasite measurements were taken both longitudinally and transversely, with means of 135.90 ± 24.18 μm and 113.70 ± 24.18 μm, respectively, at 3 h *in vitro*, 216.60 ± 18.67 μm and 132 ± 22.53 μm at 24 h p.i., and 665.70 ± 128.20 μm and 411.80 ± 47.25 μm at 8 days p.i., as illustrated in Supplemental Fig. S1.

### Protein Identification and Quantification by SWATH-MS

Using a data-dependent acquisition approach, a total of 3848 unique peptides with a local FDR <1% were identified in the library derived from all the samples pooled together, representing proteins extracted from the somatic and tegumental fractions of parasites collected at 3 h post excystment *in vitro*, as well as at 24 h and 8 days p.i. in the *in vivo* model. These peptides were collectively assigned to 1644 proteins, characterized by the presence of a minimum of two unique peptides in all samples. Of these, 1180 proteins belonged to *F. hepatica*, 444 to *M. musculus*, and 20 were found in the contaminant database. Only the parasite proteins, comprising 71.78% of the total quantified proteins, were considered for further analysis. Details of the identified proteins are provided in Supplemental Table S1.

**Fig. 2:**
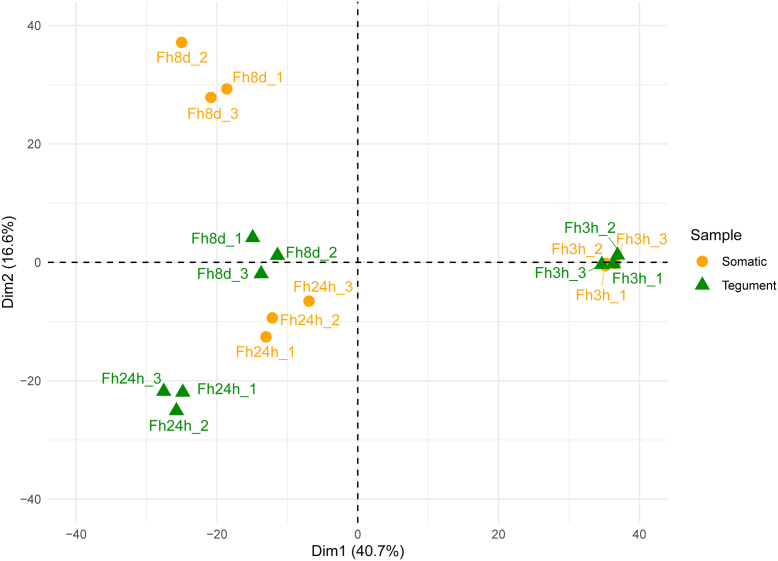
**Principal component analysis (PCA).** Positively correlated variables are grouped together, and negatively correlated variables are positioned on opposite sides of the origin of PCA. “Dim” refers to dimensionality reduction through linear mapping of data to a lower-dimensional space, maximizing the variance of the data in the low-dimensional representation. Plot displays the distribution of three replicates from 3 h *in vitro* FhNEJ (Fh3h), peritoneal cavity (Fh24h), and liver (Fh8d) from somatic (*yellow*) and tegument (*green*) proteins.

**Supplemental Fig. S2:**
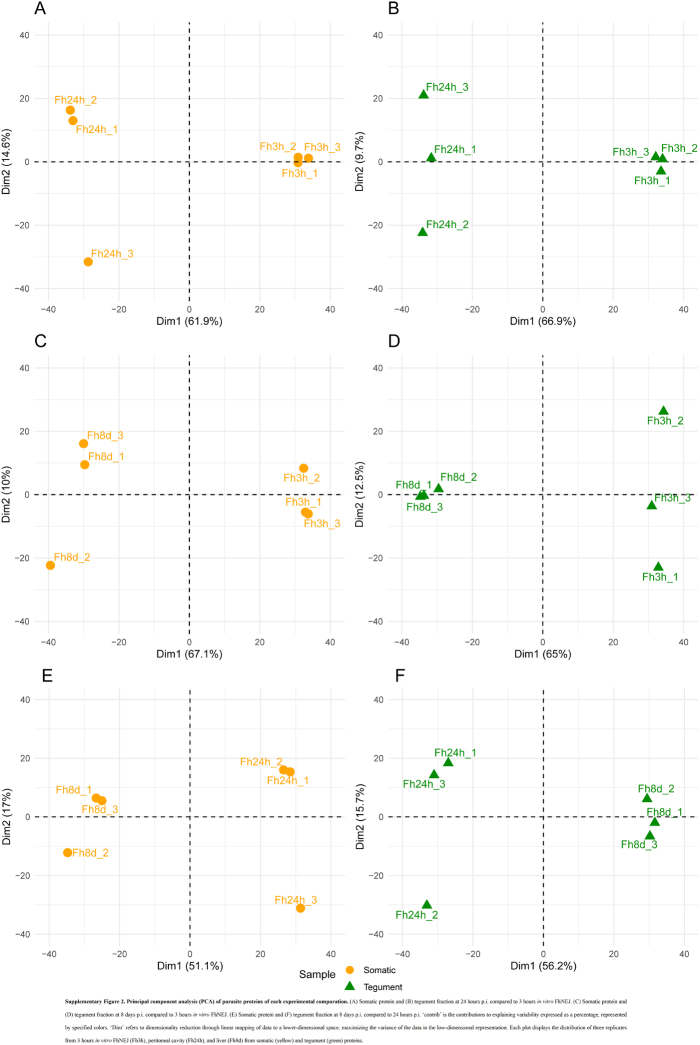


Good reproducibility was observed among the replicates and a clear segregation between proteins from the parasites obtained in the *in vivo* infection model and the FhNEJ cultured *in vitro* was demonstrated by visualizing the high-dimensional data of parasite somatic and tegument proteins in a lower-dimensional space by PCA (Fig. 2; Supplemental Fig. S2).

### Differential Expression Profile of *F. hepatica* Proteins During the Early Stages of Infection

**Fig. 3:**
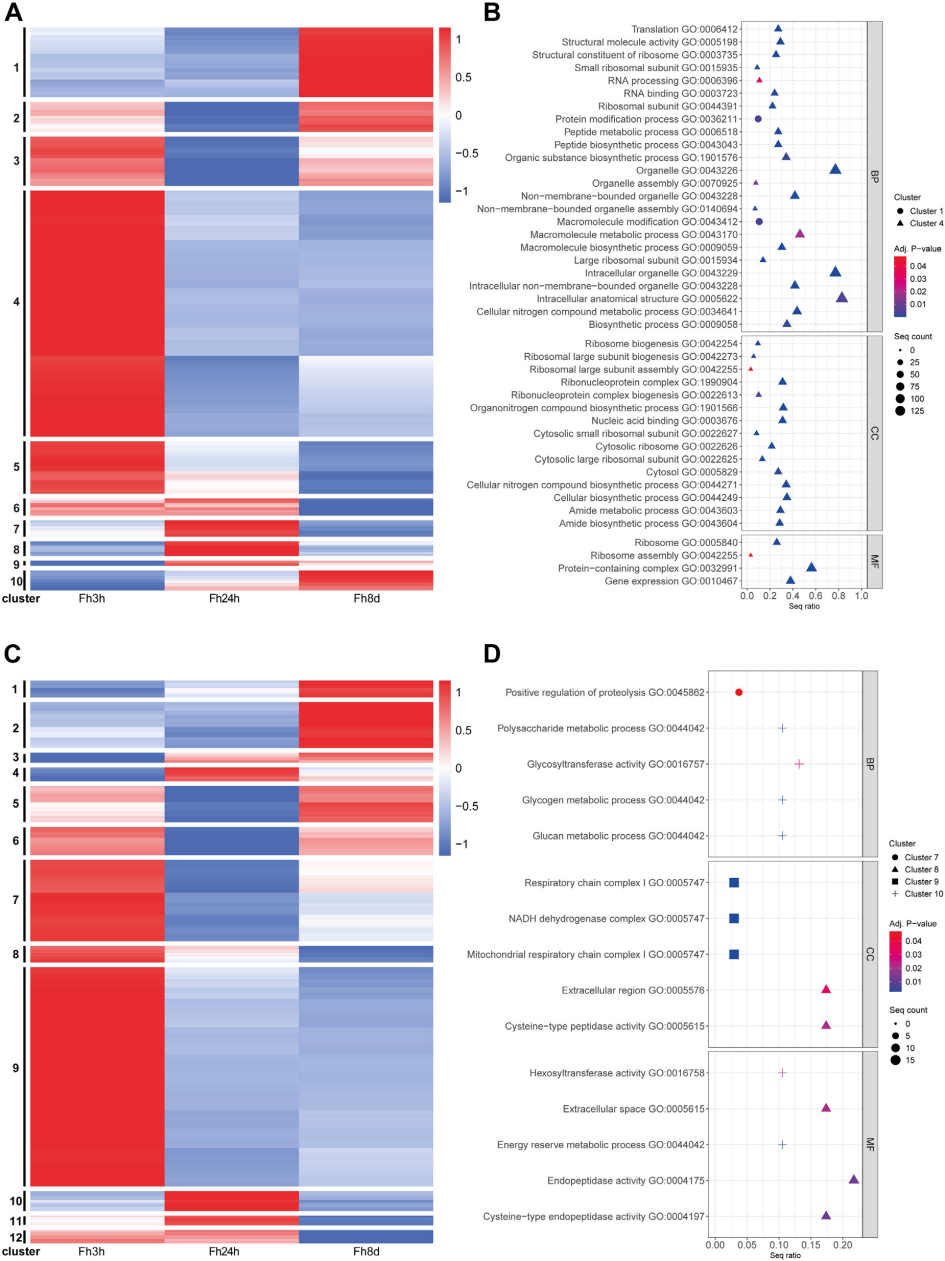
**Analysis of somatic and tegumental protein abundance and enriched gene ontology terms (GO).** Heatmap displaying hierarchical clustering of the 1180 identified proteins, depicting protein abundance patterns in (*A*) somatic and (*C*) tegument proteins (*rows*) along the parasite migration route (*columns*). The values shown are normalized results derived from SWATH-MS analysis (*red*, high abundance; *blue*, low abundance), where each column represents the mean abundance values from the three proteomic replicates. Each distinct cluster is visually highlighted by a unique color in the accompanying legend. (*B*) Somatic and (*D*) tegument GO terms classified as biological process (BP), molecular function (MF), and cellular component (CC) of enriched clusters (Fisher’s Exact Test) according to sequence ratio of somatic or tegument proteins. “Adj *p*-value” is the enrichment value for the GO term. “Seq count” is the number of protein sequences enriched for the GO term. “Seq ratio” is the percentage of total sequences in the given GO term. SWATH-MS, Sequential Window Acquisition of All Theoretical Mass Spectra.

Hierarchical clustering based on SWATH-MS derived abundance values revealed distinct clusters corresponding to the different *F. hepatica* stages analyzed: 3 h *in vitro* FhNEJ, and parasites recovered from the mouse peritoneum and liver at 24 h and 8 days p.i., as well as between parasite somatic and tegument proteins, highlighting the different localization of proteins within these extracts (Fig. 3, *A* and *C*; Supplemental Table S1). As expected, the parasites maintained for 3 h *in vitro* displayed a different protein profile to their *in vivo* counterparts. The clusters associated with the somatic proteome of the 3 h *in vitro* FhNEJ showed significant GO enrichment for terms related to macromolecular modification (GO:0043412) and protein modification process (GO:0036211), which included phosphatases and ubiquitin ligases (Fig. 3*B*). In contrast, within the tegumental fraction of the 3 h *in vitro* FhNEJ, GO enrichment was observed for terms associated with aerobic metabolism, specifically the respiratory chain complex (GO:0005747/GO:0045271), and proteins involved in glycogen metabolism. Proteins related to the regulation of proteolysis (GO:0045862) and glycan turnover (glycosyltransferase activity, GO:0016757) were also enriched in this extract (Fig. 3*D*).

The protein clusters associated with the parasites recovered from the mouse peritoneum at 24 h p.i. were relatively small compared to the other parasite stages analyzed in the study. This reflected the lack of GO enrichment in the somatic proteins and highlighted that the proteins expressed at this stage are also shared with the other parasite stages at 3 h *in vitro* and 8 days p.i. (Fig. 3*A*). However, GO enrichment was observed for cysteine-type endopeptidase activity (GO:0004197) in the tegumental fraction at 24 h p.i. (Fig. 3*D*).

**Fig. 4:**
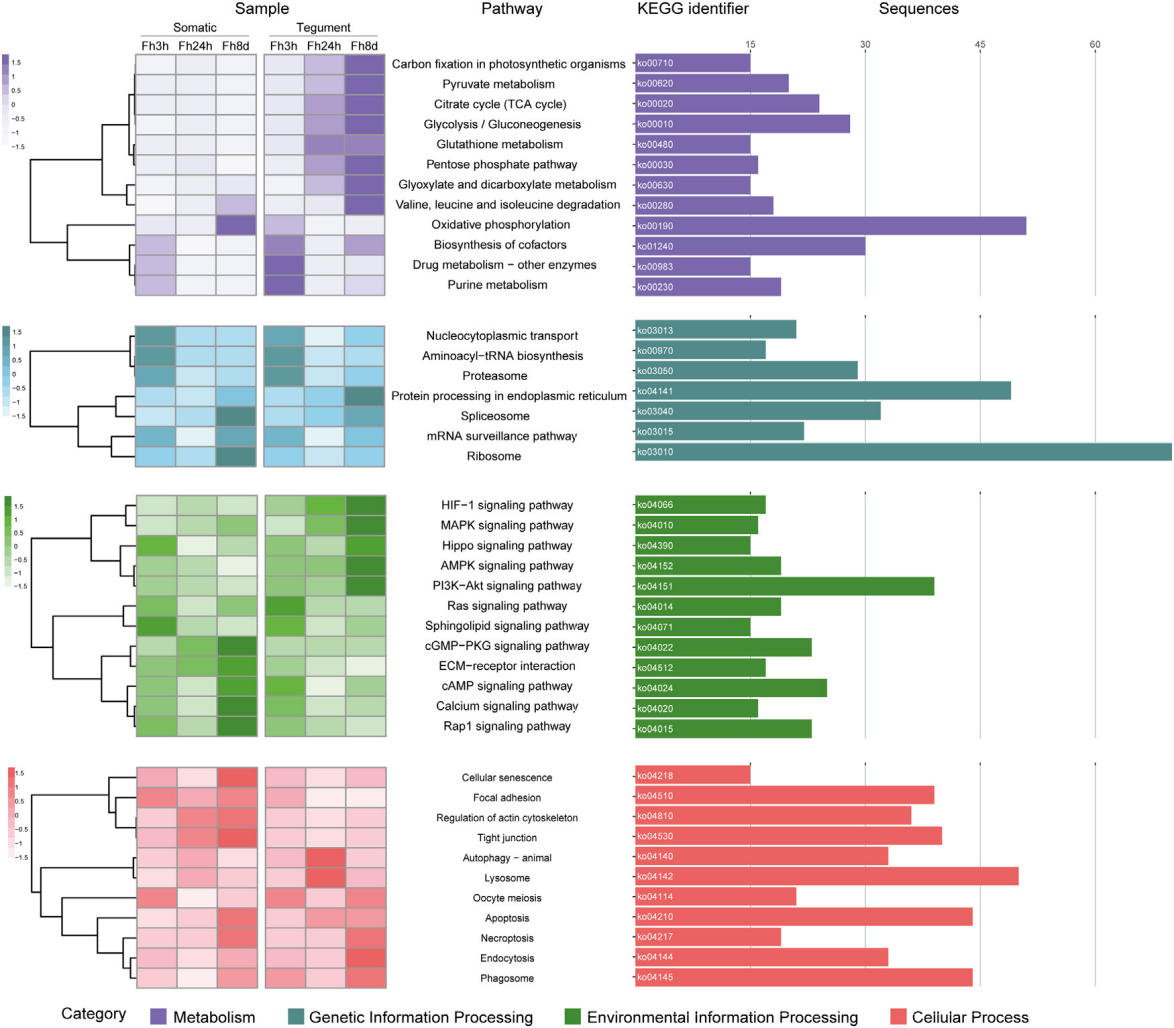
**Analysis of the Kyoto Encyclopedia of Genes and Genomes (KEGG) pathways.** The *left section* displays the average proteomic values for each sequence involved in the pathways along the parasite migration route; the first three squares depict the somatic proteins in 3 h *in vitro* FhNEJ, 24 h p.i. and 8 days p.i. parasites, followed by the profile of the tegument protein across the same sample time points. The right section quantifies the number of sequences participating in each respective pathway. “Category” refers to the KEGG pathway collection, which includes metabolism (*purple bars*), genetic information processing (*blue bars*), environmental information processes (*green bars*), and cellular processes (*orange bars*). p.i., post infection.

Indicative of increased transcriptional and translational activity, several GO terms associated with RNA processing and protein synthesis were observed in the somatic proteome of parasites recovered from the mouse liver at 8 days p.i. (Fig. 3*A*). This was also reflected in the KEGG analysis (Fig. 4, Supplemental Table S2). The clusters associated with the *F. hepatica* liver stage within the tegumental proteome did not show any specific enrichment (Fig. 3*D*) but included proteases and antioxidant proteins.

**Fig. 5:**
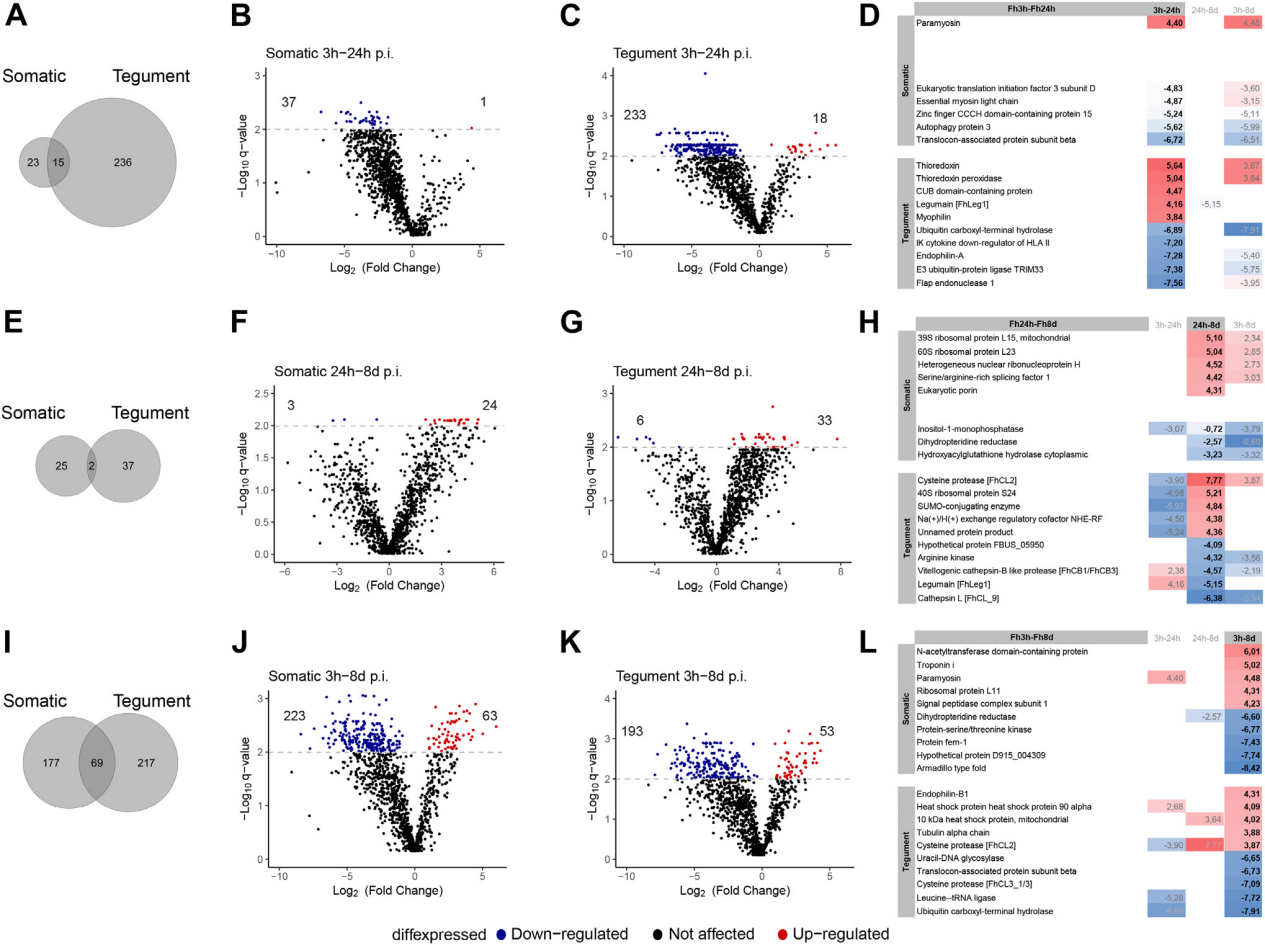
**Volcano plots illustrating the significant differential expression of proteins (DEPs) and table of DEPs in somatic and tegumental extracts.** The figure presents DEPs at three different time points: (*A*–*D*) 24 h p.i. compared to 3 h *in vitro* FhNEJ, (*E*–*H*) 8 days p.i. compared to 24 h p.i., and (*I*–*L*) 8 days p.i. compared to 3 h *in vitro* FhNEJ. For each comparison, Venn diagrams (*A*, *E*, and *I*) illustrate the overlap of DEP in somatic and tegument fractions based on accession number associated with identified proteins. Volcano plots display DEP in the somatic (*B*, *F*, and *J*) and tegument (*C*, *G*, and *K*) fractions. Proteins showing upregulation, indicating increased expression levels, are represented with *red dots*, while those showing downregulation, indicating decreased expression levels, are represented with *blue dots*. Number of DEPs are also indicated. The *gray dashed line* indicates the threshold for the identification of differentially expressed proteins (q value < 0.01). And tables (*D*, *H*, and *L*) highlight the top DEP in both fractions. The columns represent the Log2 fold change of each top protein, along with the values from the different time points, and the value is visualized using a gradient color scale ranging from *red* to *blue*, denoting high or low differentially expression, respectively. DEP, differentially expressed protein.

Focusing on the differential expression between the three parasite stages further highlighted that *F. hepatica* progressively alters the proteins it expresses depending on the host environment. Although the differential expressed proteins identified within the somatic and tegumental extracts (Fig. 5, *A*, *E* and *I*) did not overlap significantly, DEPs were observed based on the parasite stages (reflected in Supplemental Table S3). Comparative analysis of the 3 h *in vitro* FhNEJ and 24 h p.i. *in vivo* parasites (Fig. 5, *A*–*D*) revealed a total of 274 DEPs, with the majority being downregulated in the 24 h p.i. parasites with 37 somatic proteins (Fig. 5*B*) and 233 tegumental proteins (Fig. 5*C*). One protein, paramyosin, was significantly upregulated in the somatic proteome of the 24 h p.i. parasites (Fig. 5, *B* and *D*), compared with 18 proteins, including antioxidants (thioredoxin and peroxiredoxin), cysteine peptidases (cathepsins and legumains), metabolic enzymes, and structural proteins, that were significantly upregulated in the tegumental extract (Fig. 5, *C* and *D*). In contrast, the comparison between parasites obtained at 24 h and 8 days p.i. identified (Fig. 5, *E*–*H*) a lower number of DEP (64 DEPs), with the majority being upregulated proteins in the 8 days p.i. parasites with 24 somatic proteins (Fig. 5*F*) and 33 tegumental proteins (Fig. 5*G*). The significantly upregulated somatic proteins in this extract were primarily associated with the ribosome, indicative of increased transcription and translation activity occurring at 8 days p.i. Proteins involved in these functions (ribosomal proteins, ADP-ribose, Histone H3-lysine (27) N-trimethyltransferase) were also upregulated within the tegumental protein fraction at this parasite stage, along with cathepsin L peptidases, heat shock proteins, and proteins involved in metabolism (Fig. 5*H*).

As expected, the greatest differential expression between *F. hepatica* stages was observed between the 3 h *in vitro* FhNEJ and 8 days p.i. *in vivo* parasites (Fig. 5, *I*–*L*). In the somatic proteome of the liver stage parasites, 286 DEPs were identified, representing 63 upregulated and 223 downregulated proteins (Fig. 5*J*), compared to the 246 DEPs in the tegumental protein fraction, with 53 upregulated and 193 downregulated proteins (Fig. 5*K*). This profile reflected the vast differences between the 3 h *in vitro* FhNEJ (∼135.9 μm in length), and the more developed 8 days p.i. parasites (∼665.7 μm in length) (Supplemental Fig. S1). Similar to the proteins identified in the 24 h versus 8 days p.i. parasite stages comparison, key proteins of interest included those involved in transcription/translation, metabolism, cathepsins, legumains, glutathione S transferases, and protease inhibitors (Supplemental Table S3).

### *F. hepatica* Metabolic Pathways During Early stage Host Migration

**Fig. 6:**
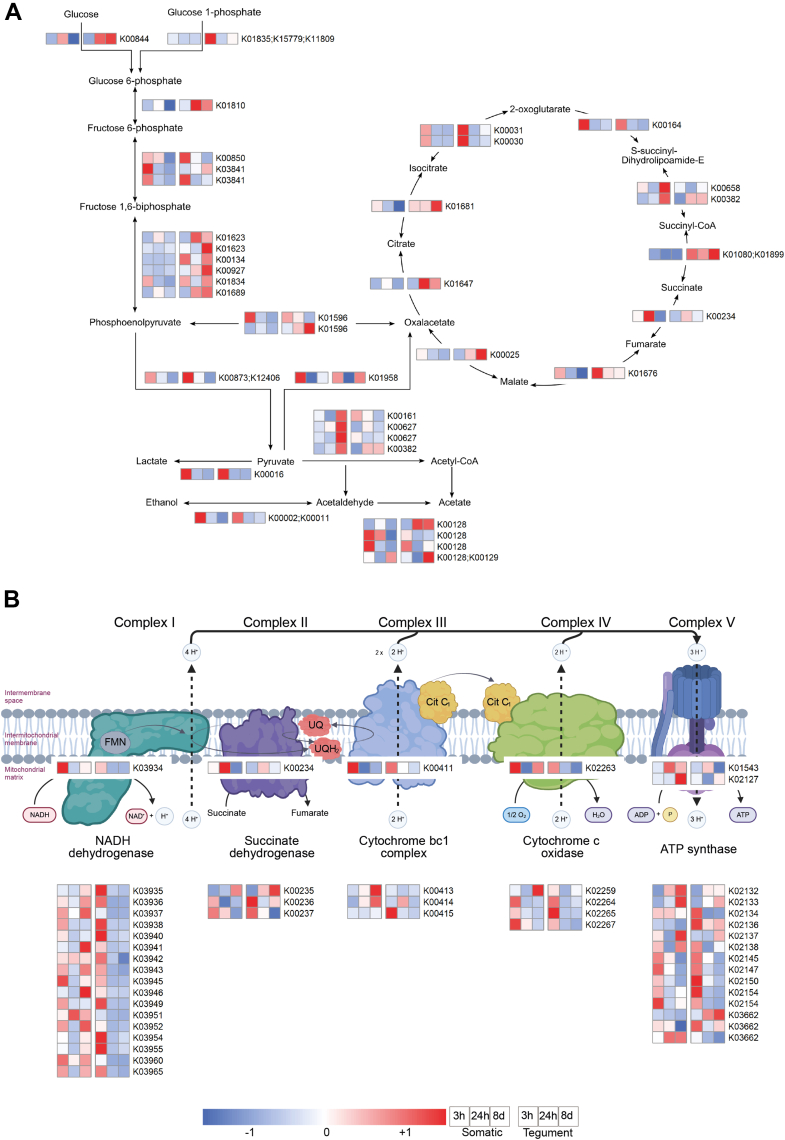
**Heatmaps of the comparative proteomic expression profiles of energy metabolism KEGG pathways in parasites at different times post infection.***A*, glycolysis, tricarboxylic acid cycle, lactate, and acetate pathways. *B*, oxidative phosphorylation pathway. The first three squares correspond to the 3 h *in vitro* FhNEJ, 24 h p.i. and 8 days p.i. parasites for somatic proteins, respectively, while the remaining three squares represent the same parasites samples for tegument proteins. Expression levels are visualized using a gradient color scale ranging from *blue* to *red*, denoting low to high expression, respectively. Based on Cwiklinski *et al*. (2018) (13).

To analyze the metabolic pathways associated with the early migration of *F. hepatica* in the established *in vivo* model, we studied the KEGG pathways related to energy metabolism, based on the KEGG orthologs identified in our proteome. In this way, we observed increased expression of the pathways involved in aerobic metabolism, particularly the glycolysis and gluconeogenesis routes, the citrate/TCA cycle, and the oxidative phosphorylation pathway, which includes the respiratory chain complex (Fig. 6). No distinct pattern was observed for the glycolysis/TCA pathways between the different parasite stages and protein extract types (Fig. 6*A*). However, a subtle shift toward anaerobic metabolism was observed in the 8-day liver stage parasites, particularly in the tegumental fraction, including 1) decreased expression of pyruvate kinase (K00873) and succinate dehydrogenase (K00234), and 2) increased expression of malic enzyme (K00025) and the glyoxylate and dicarboxylate metabolism pathway (Fig. 6*A*).

In contrast to the glycolysis/TCA pathways, distinct protein expression was observed for the oxidative phosphorylation route (Fig. 6*B*). Specifically, the majority of these proteins were highly expressed within the tegumental fraction, predominantly for the 3 h *in vitro* FhNEJ stage. The somatic proteins were more comparable for the different parasite stages, with only a few proteins displaying higher levels of expression, predominantly in the liver associated parasite stages.

Regarding signal transduction pathways involved in parasite growth and development, our data show an upregulation of these routes at 8 days p.i., indicating their activation once the parasite reaches the liver. These include 1) pathways associated with the generation and proliferation of pluripotent cells (PI3K-Akt and MAPK signaling pathway), 2) regulation of cellular growth (AMPK and Hippo signaling pathways), and 3) regulation of oxygen-related metabolic processes (HIF-1 signaling pathway) (Fig. 4).

### Differential Expression of Key *F. hepatica* Cysteine Peptidases and Protease Inhibitors

**Fig. 7:**
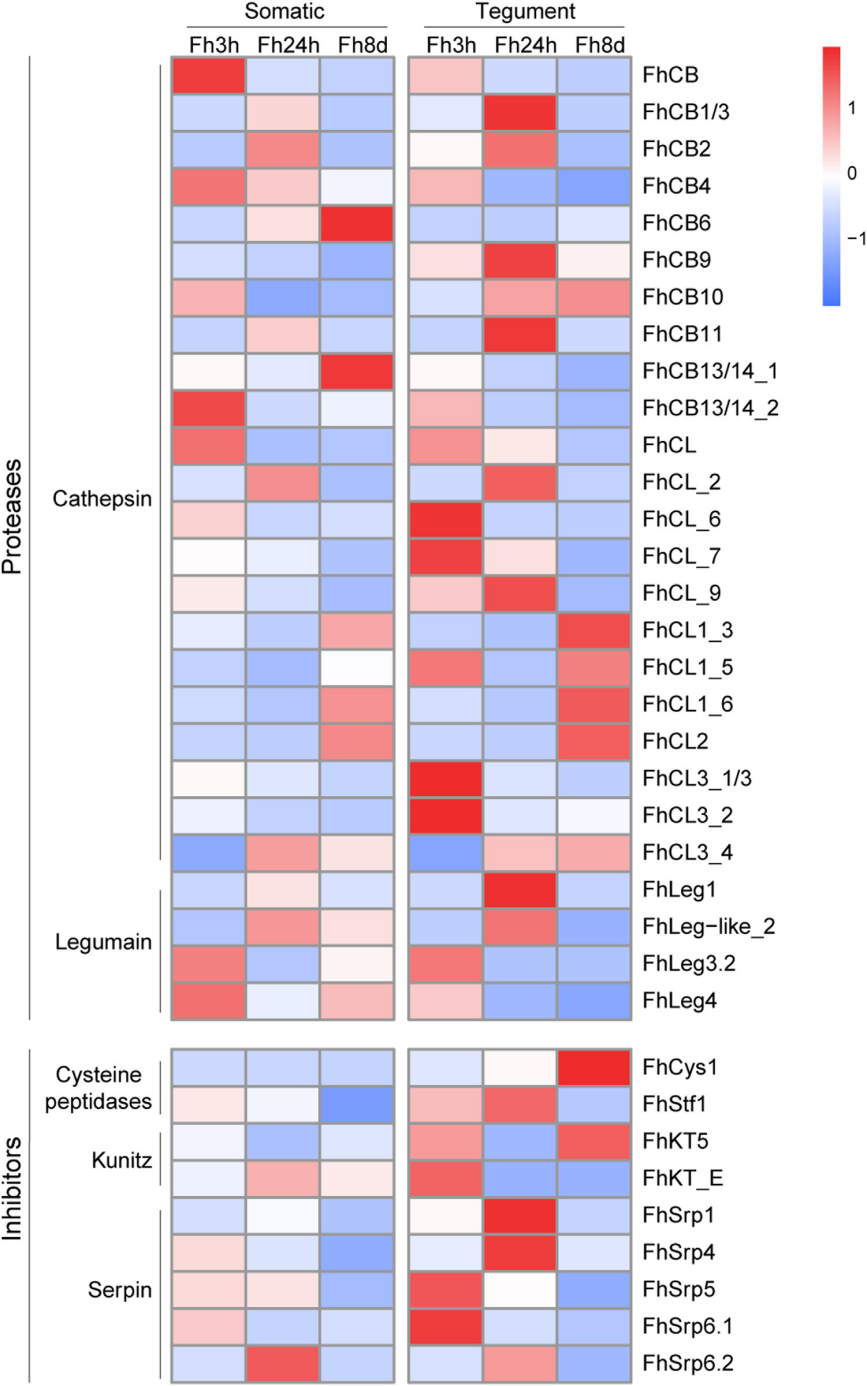
**Heatmap displaying the expression profile of key cysteine peptidases and their inhibitors.** The plot displays the proteomic values of each annotated protein, organized by their respective families. Proteases are categorized as cathepsin and legumain in the *upper* block, while the protease inhibitors are categorized by the peptidase type they inhibit, namely as cysteine peptidases (cystatin and stefin), kunitz and serpin in the *lower* block. The first three squares depict the 3 h *in vitro* FhNEJ, 24 h p.i. and 8 days p.i. parasites for somatic proteins, *while* the subsequent three squares illustrate the same samples for tegument proteins. Expression levels are represented visually with a gradient color scale that ranges from *blue* to *red*, indicating low to high expression, respectively.

Next, we conducted an analysis of the differential expression of various families of cysteine peptidases and protease inhibitors due to their proven involvement in the migration and survival processes of *F. hepatica* within its mammalian host. Our analysis highlighted that an abundance of cathepsin peptidases was expressed by the three parasite stages, with a specific enrichment within the tegumental protein fraction (Figs. 3*D*, 5, 7). Particularly, we observed a distinct profile of cathepsin peptidases within the 3 h FhNEJ somatic proteome, with notable abundance of cathepsins B4, B10, and B13/14, in addition to several proteins putatively identified as cathepsin B and cathepsin L peptidases, which have yet to be fully characterized. On the other hand, other cathepsins of the B type, namely cathepsins B1, B2, and B3, were more highly expressed by the 24 h parasite stages, within both the somatic and tegumental protein fractions. Comparable results were observed for the cathepsin L3 peptidases and legumain 1, which display higher levels of expression at 24 h p.i., highlighting the role these peptidases play in the migratory stages of infection. As expected, a different profile of cathepsin peptidases is expressed by the parasites obtained at 8 days p.i., encompassing a range of the peptidases associated with both the early and adult stage parasites. The somatic proteome of the 8 days p.i. parasites displayed an abundance of cathepsins B6 and B13/B14, whereas, in contrast, the tegumental protein fraction of this stage, expressed an abundance of cathepsin L peptidases, including CL1, CL2, and CL3 type peptidases (Fig. 7).

Secondly, regarding protease inhibitors, with the exception of peptides mapping to serpin 6 (FhSrp6) that were expressed within the somatic proteome of the 24 h p.i. stage parasites, we observed higher levels of expression of the parasite inhibitors within the tegumental protein fractions (Fig. 7). Predominant expression of the cystatins/stefins, namely the stefin 1 (FhStf1) and the multidomain cystatin (FhCys1), was evident for the peritoneum and liver associated parasite stages, respectively. Two Kunitz-type inhibitors (FhKT5 and FhKT_E) were identified in our dataset that were expressed by the 3 h *in vitro* and 8 days p.i. parasites. Finally, the liver associated parasite stages in our study did not express an abundance of serpins, with higher levels of expression observed in the 3 h *in vitro* (FhSrp5 and FhSrp6) and 24 h p.i. (FhSrp1 and FhSrp4) parasite stages (Fig. 7). The proteases and inhibitors identified by manual annotation in the different parasite stages in this study and their abundance are shown in the Supplemental Table S4.

### Validation of Manual Proteomic Annotation

To validate the manual proteomic annotation of the FhCB3 protein, tegumental extracts from both 3 h *in vitro* FhNEJ and 8 days p.i. parasites were subjected to SDS-PAGE and immunoblotted using an antibody against FhCB3. Reactivity was detected at ∼37 kDa as a zymogen and ∼28 kDa as the mature enzyme domain of FhCB3 in the tegumental extract of 3 h *in vitro* FhNEJ, whereas low reactivity was detected in the same extract from liver associated parasite stages (Supplemental Fig. S3), as expected based on the abundance results obtained for this protein in our proteomic study (Supplemental Table S4).

## Discussion

Tissue migration in helminth parasites represents a selectively advantageous strategy despite its high energy cost and the need for adaptation to the changing physiological environment (35). Using this strategy, these parasites implement mechanisms to evade the immune response in the host’s compartments while they continue to grow and develop (35). In fasciolosis caused by *F. hepatica*, migration involves two key stages: a rapid passage through the intestinal wall into the peritoneal cavity, limiting parasite exposure to the gut immune system (9, 14), and burrowing through the liver tissue, allowing parasite development while evading immune responses (36) until the final migration to the bile ducts.

To investigate the changes that this helminth parasite undergoes during the early stages of invasion/migration, we used a SWATH-MS proteomic approach on samples recovered from an *in vivo* mouse *F hepatica* infection model during the first 8 days of infection. Our analysis of the 8-day parasites is the first investigation of this time point using proteomic tools, contributing to our knowledge of liver stage parasites during the early infection. In comparison to other proteomics techniques, SWATH-MS integrates both data-dependent and data-independent acquisitions, thereby enhancing both quantification and data acquisition (37, 38). This approach enabled the identification of a greater number of parasite proteins compared to previous proteomic studies of *F. hepatica*, including more than twice the number of proteins identified in the *ex vivo* model of the mouse intestine used to study parasite migration across the intestinal barrier (488 proteins) (14). It also exceeded the 689 proteins reported for FhNEJ and adult worm stages in the study published by Di Maggio *et al*. (2016) (18), thereby enhancing our understanding of early stage parasite development.

Recently, there has been an increase in the development of *in vitro* models for *F. hepatica* studies (13, 14, 39, 40). Providing a comprehensive view of parasite development during the first stages of infection will be crucial in determining how representative these *in vitro* models are. Recovery of *F. hepatica* parasites from ruminant hosts, particularly early stage parasites, is complicated by the disproportionate ratio of parasites to host tissue size, often resulting in low recovery rates. In this study, we used a C57BL/6 mouse infection model to investigate the optimal time points for maximizing parasite recovery for the early stage parasites found in the peritoneum and liver. Focusing on two time points, 24 h and 8 days p.i., we substantially increased parasite recovery rates, which were suitable for downstream proteomic analyses. Our recovery rates are comparable to those reported by Tkalcevic *et al*. (1996) (18) in a previous study conducted with BALB/c mice using a similar model. Interestingly, our data, combined with our previously developed *ex vivo* model of parasite migration across the intestine (13), highlight that only a small proportion of FhNEJ (approximately 22% of an infective oral metacercariae dose or FhNEJ injection) are capable of efficiently migrating through the intestinal wall and to continue their journey toward the liver.

In this study, we compared two compartments of the parasite: the somatic fraction to investigate parasite development, and the tegumental fraction, which is considered a potential source of therapeutic targets against fasciolosis (9). The resulting proteins from these two extracts displayed distinct profiles. Specifically, the tegumental fraction displayed greater enrichment for proteins involved in metabolism and cysteine peptidases, including cathepsin peptidases and legumains, which are known to play an important role in the liver fluke life cycle and have been previously described for this parasite compartment (9) (Fig. 3*D*). The significant abundance of key proteins of interest may have been a result of selectively enriching for the tegumental proteins, similar to what was observed in the study by Ravida *et al*. (2016) (41) that investigated the glycoproteins within the adult *F. hepatica* tegument.

The *F. hepatica* tegument has been previously described as being metabolically active (42). In agreement with those observations, proteins involved in metabolism, particularly aerobic metabolism and signal transduction pathways, were identified in both the somatic and tegumental compartments. It has been previously described that the early stage *F. hepatica* parasites undergo aerobic metabolism due to their size and the fact that oxygen can permeate across the tegumental wall (10). In line with previous studies, we identified proteins involved in pathways related to glycolysis, TCA/citrate cycle, and oxidative phosphorylation (Supplemental Table S2). Our results were comparable to the proteomic study by Cwiklinski *et al*. (2018) (13), who described significant changes in the expression of key glycolytic enzymes during the early stages of *F. hepatica* at 1 h, 3 h, and 24 h post excystment. Although we observed a decrease in enzymes such as glucose-6-phosphate isomerase (K01810), 6-phosphofructokinase 1 (K00850), and pyruvate kinase (K00873) between 3 h *in vitro* and 24 h p.i., the Cwiklinski *et al*. (2018) (13) study highlighted the dynamic regulation of glycolysis and glycogen metabolism to meet the energy demands associated with early infection. Once the parasites reach the liver, a metabolic shift begins, relating to acetate production under oxygen-poor conditions, such as those found in the liver. This shift is marked by increased expression of enzymes that facilitate the conversion of pyruvate to acetyl-CoA, which is then converted to acetate via aldehyde dehydrogenase (K00128 and K00129).

Regarding signal transduction pathways, these routes have been shown to be associated with parasite growth and development in an *in vivo F. hepatica* infection model, in which 21-day immature flukes were extracted from the liver of BALB/c mice by Cwiklinski *et al*. (2021) (17). Our results demonstrate that the intense cellular differentiation that drives parasite growth begins as *F. hepatica* reaches the liver, with abundant expression of the same signal transduction routes at 8 days p.i., including PI3K-Akt, AMPK, and Hippo signaling pathways, which regulate cell growth and proliferation, as well as the HIF-1 signaling pathway, which regulates oxygen homeostasis and is consistent with the switch to more anaerobic metabolic processes. Similarly, GO terms associated with RNA processing and protein synthesis, indicators of extensive transcriptional/translational activity, were observed in the *F. hepatica* flukes obtained from both *in vivo* models at 8 days p.i. (corresponding to the present study) and 21 days p.i. (in the study by Cwiklinski *et al*. (2021)) (17). This further underscores the role of the host microenvironment in driving parasite development.

It has been previously shown that the *F. hepatica* cathepsin peptidases are released into the host environment by secretion from gastrodermal cells within the blind-ended gut, consistent with their role in tissue and blood feeding (11). Here, enrichment of the cysteine peptidases was observed within the tegumental protein fraction. This may be explained by the fact that these proteins form a large component of ES products secreted by the *F. hepatica* life cycle stages, which may adhere to the outer tegumental surface following secretion. Analyses of the *F. hepatica* ES products have also shown that, in addition to soluble molecules, proteins are released by the parasite encapsulated in extracellular vesicles (EVs) (43). Cathepsin peptidases have been identified within the adult *F. hepatica* EVs (28), and while studies have shown that the majority of EVs in adult parasites are released from the gut surface, the tegument also plays a role in their secretion (44). This implies that a similar process may occur for the early stage parasites, resulting in the cysteine peptidase enrichment we observed. The role EVs is further supported by the upregulated endophilin-B1 in the tegumental protein fraction at 8 days p.i., which is known to be involved in membrane dynamics and vesicle trafficking.

Differential expression analysis of the cathepsin peptidases revealed a dynamic profile comparable to that previously described (11, 45). In this regard, FhCB1, FhCB2, and FhCB3 displayed peak expression at 24 h p.i., which decreased by 8 days p.i. in our *in vivo* model. Similarly, these peptidases have been found in the secretions of FhNEJ after 24 h of *in vitro* incubation in the study by Cwiklinski *et al*. (2018) (13), highlighting their early role in the biology of FhNEJ. In contrast, FhCB4 maintained consistent expression levels throughout the infection period, while the other cathepsin B peptidases (FhCB6, FhCB13/14_1) displayed increased expression once the parasites reached the liver, consistent with analysis of these peptidases throughout the life cycle (11). Cathepsin L3 peptidases (including FhCL3_1/3 and FhCL3_2) were predominantly expressed by the parasites obtained *in vitro* at 3 h, as also observed in those analyzed at 24 h after excystment in the study by Cwiklinski *et al*. (2018) (13). On the other hand, the parasites obtained at 8 days p.i. showed a more dynamic profile consisting of multiple cathepsin L clades, namely FhCL1 and FhCL2, reflecting a change in feeding behavior, while also overexpressing FhCL3_4. The expression of both the collagenolytic CL3 peptidases and the hemolytic CL1 peptidases is consistent with that previously observed for the 21-day immature flukes (17), highlighting that these liver migratory stages are capable of feeding on both tissue and blood.

In depth analyses of the *F. hepatica* genome have facilitated our analyses of the various gene families important for the parasite, including the cathepsin peptidases. Although functional characterization has been carried out on several members of this family, more biochemical analyses are required to determine the function of some of the newly identified members, to further elucidate the role they play during the *F. hepatica* life cycle. The current version of the *F. hepatica* genome, housed at WormBase ParaSite (https://parasite.wormbase.org/Fasciola_hepatica_prjeb58756/Info/Index/), is comprised of mainly 10 large scaffolds, representing the 10 *F. hepatica* chromosomes. This version of the genome now facilitates further molecular characterization of this important gene family to identify all the various members and the role they play throughout the *F. hepatica* life cycle.

*F. hepatica* expresses a range of cysteine peptidase inhibitors to control and manipulate both parasite and host cathepsin peptidases, including several cystatins/stefins and purposively adapted Kunitz-type inhibitors (13). Our proteomic analyses identified an abundance of stefin 1 (FhStf1) at the 3 h *in vitro* and 24 h p.i. parasite stages, consistent with this inhibitor being shown to be active against both native cysteine peptidases in the FhNEJ ES products and recombinant versions of FhCL1, FhCL2, and FhCL3 (46). Conversely, the multidomain cystatin (FhCys1) was more highly expressed by the parasites recovered from the liver; however, the exact function and specificity of this protein is unknown, limiting our understanding of its role in the liver stage parasites.

In contrast to other *F. hepatica* proteomic studies, the Kunitz-type inhibitors from the group FhKT1, which have been found in the parasite secretomes of both FhNEJ and adult worms (47), were not identified in our study. Instead, FhKT5 was expressed in the tegumental protein fraction, particularly after the parasite reached the liver. FhKT5 has not been previously identified in the *F. hepatica* somatic proteomes; however, our results complement the transcriptome data described by Smith *et al*. (2020) (47), which showed increased transcription in immature flukes obtained at 21 days p.i. Both FhKT1 and FhKT5 inhibitors contain a leucine residue in their active site, indicating that they may have comparable function, specifically the inhibition of cysteine peptidases (48).

In addition, while not secreting any serine peptidases, *F. hepatica* expresses and secretes a family of serine peptidase inhibitors, serpins (49). Our proteomic data also included several key members of the *F. hepatica* serpin family, specifically four serpins (FhSrp1, FhSrp4, FhSrp5, and FhSrp6) in both the somatic and tegumental proteomes. Although the exact function of all the serpin members is currently unknown, several members have been shown to display inhibitory properties against a range of mammalian serine peptidases, including kallikrein, chymotrypsin, and cathepsin G (18, 49). FhSrp1 and FhSrp2 have also been shown to be involved in inhibiting activation of the complement via the Lectin pathway (50). Our proteomic data complement the transcriptome data, highlighting increased expression of these inhibitors by the early stage parasites (3 h *in vitro* and 24 h p.i. parasites) in comparison to that observed once the parasites have reached the liver (49). Further functional analyses of these serpins are required to determine their role during the *F. hepatica* life cycle and why these are not important once the parasite reaches the liver.

We identified several other proteins that were also found abundantly expressed in other proteomic studies, highlighting their importance during the early stages of fasciolosis. At 24 h and 8 days p.i. stages, paramyosin was upregulated in the *F. hepatica* somatic proteome. This is consistent with previous analyses that described an abundance of this protein within the FhNEJ and 21-day immature fluke somatic proteomes (13, 17). Although not abundantly expressed within the tegumental protein fraction, this protein could still be exposed to the host environment, as it can also be found within the secreted protein fractions (13, 17), where it may play a role in the inhibition of the classic and alternative complement pathways (51).

Finally, the antioxidants thioredoxin (FhTrx) and peroxiredoxin (FhPrx) were also abundantly expressed in our proteomes particularly in the 24 h and 8 days p.i. stages. These antioxidants protect the parasite against oxidative stress. In addition, these proteins could play moonlighting immunomodulation roles following their secretion by *F. hepatica* (52). In particular, FhPrx triggers a Th2-type response (53). The abundant expression of these antioxidants in the parasites obtained at 8 days p.i. is consistent with that observed in the 21-day immature parasites in the study by Cwiklinski *et al*. (2021) (17), where both FhTrx and FhPrx were identified in the somatic proteome and secretome, indicating an important role for these molecules in the liver-associated stages.

In this study, we successfully recovered a sufficient number of *F. hepatica* parasites from the early infection stages using an optimized *in vivo* model. Combined with the proteomic SWATH-MS technique, this allowed us to generate the first proteomic data from *in vivo*-derived parasites at 24 h and 8 days p.i., when the parasites reach the peritoneum and liver, respectively, within the rodent model. Interestingly, the 8-day p.i. stage showed several proteins previously identified in 21-day immature flukes recovered *in vivo*, revealing early changes induced by the host on the parasite. These findings highlight the importance of host–parasite interactions in driving parasite development and suggest that these processes begin earlier than previously thought. Encouragingly, our results are mainly consistent with other *F. hepatica* proteomic studies, reinforcing the key proteins involved in early invasion and infection. The consistent identification of these proteins across both *in vitro* and *in vivo* studies suggests that *in vitro* models can support parasite development. Moreover, the incorporation of tissue-specific cells into these models could enhance their relevance by promoting appropriate host–parasite signaling, making them valuable for future studies of early stage infection in fasciolosis.

## Data Availability

The mass spectrometry proteomics data have been deposited to the ProteomeXchange Consortium via the PRIDE (20) partner repository with the dataset identifier PXD059684.

## Supplemental data

This article contains supplemental data.

## Conflict of Interest

The authors declare no competing interests.

## Figures and Tables

**Supplemental Fig. S3:**